# Variation at interleukin-6 receptor gene is associated to joint damage in rheumatoid arthritis

**DOI:** 10.1186/s13075-015-0737-8

**Published:** 2015-09-04

**Authors:** Maria Lopez-Lasanta, Antonio Julià, Joan Maymó, Benjamín Fernández-Gutierrez, Inmaculada Ureña-Garnica, Francisco J. Blanco, Juan D. Cañete, Mercedes Alperi-López, Alex Olivè, Héctor Corominas, Jesus Tornero, Alba Erra, Miriam Almirall, Nuria Palau, Ana Ortiz, Gabriela Avila, Luis Rodriguez-Rodriguez, Arnald Alonso, Raül Tortosa, Isidoro Gonzalez-Alvaro, Sara Marsal

**Affiliations:** Vall d’Hebron Hospital Research Institute, Rheumatology Research Group, Pg Vall Hebron 119-129, 08035 Barcelona, Spain; Rheumatology Department, Hospital del Mar, Pg Marítim, 25-29, 08003 Barcelona, Spain; Rheumatology Department, Hospital Clínico San Carlos, C. Prof. Martin Lagos, S/N, 28040 Madrid, Spain; UGC Reumatología, Instituto de Investigación Biomédica de Málaga (IBIMA), Hospital Regional Universitario de Málaga, Avda. Carlos Haya, S/N, 29010 Málaga, Spain; Rheumatology Department, INIBIC-Hospital Universitario A Coruña, C. As Xubias, 84, 15006 A Coruña, Spain; Rheumatology Department, Hospital Clínic de Barcelona, C. Villaroel, 170, 08036 Barcelona, Spain; Rheumatology Department, Hospital Universitario Central de Asturias, Avda. De Roma, S/N, 33011 Oviedo, Spain; Rheumatology Department, Hospital Universitari Germans Trias i Pujol, Crta. Can Ruti-Camí Escoles, S/N, 08916 Barcelona, Spain; Rheumatology Department, Hospital Moisès Broggi, C. Jacint Verdaguer, 90, 08906, L´Hospitalet de Llobregat, Barcelona, Spain; Rheumatology Department, Hospital Universitario De Guadalajara, C. Donantes de sangre, S/N, 19002 Guadalajara, Spain; Rheumatology Department, Hospital Sant Rafael, Pg Vall Hebron, 107, 08035 Barcelona, Spain; Rheumatology Department, Hospital Universitario La Princesa, IIS La Princesa, C. Diego Leon, 62, 28006 Madrid, Spain

## Abstract

**Introduction:**

Interleukin-6 (IL-6) cytokine signaling is key in Rheumatoid Arthritis (RA) pathophysiology. Blocking IL-6 receptor (IL6R) has proven to be a highly effective treatment to prevent joint damage. This study was performed to investigate the association between the genetic variation at *IL6R* gene and the severity of joint damage in RA.

**Methods:**

*IL6R* gene tagging SNPs (*n* = 5) were genotyped in a discovery group of 527 RA patients from 5 different university hospitals from Spain. For each marker, a linear regression analysis was performed using an additive model and adjusting for the years of evolution of the disease, autoantibody status, gender and age. Haplotypes combining the SNPs were also estimated and tested for association with the level of joint destruction. Using an independent cohort of 705 RA patients from 6 university hospitals we performed a validation study of the SNPs associated in the discovery phase.

**Results:**

In the discovery group we found a highly significant association between *IL6R* SNP rs4845618 and the level of joint destruction in RA (*P* = 0.0058, *P*_*corrected*_ = 0.026), and a moderate association with SNP rs4453032 (*P* = 0.02, *P*_*corrected*_ = 0.05). The resulting haplotype from both SNPs was more significantly associated with joint damage (*P* = 0.0037, *P*_*corrected*_ = 0.011). Using the validation cohort, we replicated the association between the two IL-6R SNPs with the degree of joint destruction in RA (*P* = 0.007 and *P* = 0.04, meta-analysis *P* = 0.00011 and *P* = 0.0021, respectively), and the haplotype association (*P* = 0.0058, meta-analysis *P* = 6.64 e-5).

**Conclusions:**

Genetic variation at *IL6R* gene is associated with joint damage in RA.

**Electronic supplementary material:**

The online version of this article (doi:10.1186/s13075-015-0737-8) contains supplementary material, which is available to authorized users.

## Introduction

Rheumatoid arthritis (RA) is a systemic autoimmune disease with an estimated prevalence of ~1 % in the general population. RA is characterized by a chronic inflammation of the synovial membrane and the progressive destruction of the joint cartilage and subchondral bone. RA is a complex disease in which the interplay between multiple genetic and environmental factors determines not only its onset but also its evolution to more severe forms [1].

The extent of joint damage is a clinically relevant and highly variable feature among RA patients. Recent studies have shown that there is a genetic predisposition to develop higher levels of joint destruction in RA. The heritability of this clinically relevant trait has been estimated to range from 45 to 60 % [2]. The identification of the genes and genetic pathways that contribute to increase the risk of joint damage in RA will clearly be of high value for the future development of prognostic tests as well as for the design of more efficient therapeutic approaches.

The interleukin (IL)-6 signaling pathway is strongly associated with RA pathophysiology [3]. IL-6 cytokine is highly expressed in the inflamed synovial tissue of RA patients and influences the functionality of multiple cell types including macrophages and T and B lymphocytes and osteoclasts [4]. These effects are mediated by the binding of the cytokine to the IL-6 receptor protein (IL6R, CD126), which is expressed both on the surface of the cell membranes as well as in a soluble form. Targeting the cell-bound and soluble IL-6R using monoclonal antibodies has proven to be an efficacious therapy in RA, significantly reducing structural damage [5]. Consequently, the gene encoding IL-6R is a strong candidate for association with the level of joint destruction in RA.

We performed a candidate gene study of the association of IL-6 receptor gene (*IL6R*) with the level of joint damage in RA. To test this hypothesis we identified single nucleotide polymorphisms (SNPs) tagging the *IL6R* locus and analyzed their association in a discovery cohort of RA patients from Spain. Using an independent cohort of patients, we have subsequently validated the observed associations.

## Methods

### Study population

All RA patients recruited for this study satisfied the American College of Rheumatology diagnostic criteria for RA [6] and had >2 years of follow-up since diagnosis. Also, all patients were Caucasian with all four grandparents born in Spain. Two cohorts of patients from different hospitals in Spain were collected to identify the polymorphisms associated with joint destruction (i.e., discovery phase cohort) and to subsequently validate these SNPs (i.e., validation phase cohort).

This study was undertaken in compliance with the Helsinki Declaration. Informed consent was obtained from all participants, and protocols were reviewed and approved by local institutional review boards. Ethical approval was obtained from the Vall d´Hebron Hospital Ethics Committee.

#### Discovery and validation phase cohorts

In the discovery phase, 527 patients were recruited from five hospital centers: Hospital Universitario de Asturias, Hospital Clínic i Provincial de Barcelona, Hospital Universitari Vall d'Hebron, Hospital Universitario de Guadalajara, and Hospital del Mar. In the replication phase, RA patients were collected from six different hospitals (*n* = 705): Hospital de San Rafael, Hospital Universitario La Princesa, INIBIC-Hospital Universitario A Coruña, Hospital Clínico San Carlos, Hospital Universitari Germans Trias i Pujol, and Hospital Regional Universitario de Málaga.

#### Joint damage scoring

Clinical and epidemiological variables were collected from all patients. Hand and feet radiographic images were obtained from all patients during the inclusion period. Importantly, joint damage was quantified using the same method in all participating rheumatology departments. This S-score method is a joint damage scoring system that captures the level of joint destruction in each patient using a more practical approach compared with other, more complex methods [7, 8]. Given the large number of samples required to analyze the genetic association between *IL6R* and joint damage, this score was designed to simplify the collection of this trait while reducing the time for acquisition of data in the clinical setting. In the S-score method, six different key areas of the hands and feet are evaluated and quantified from radiographs—distal radius (0/1 points), distal ulna (0/1 points), wrist (0/1 points), metacarpophalangeal joints (0–5 points), interphalangeal joints (0–5 points), and metatarsophalangeal joints (0–5 points)—for each side and for both hands and feet. In each of these areas, the presence of an erosion is considered whenever there is evident loss in the cortical region or loss in the normal bone shape (i.e., referred to as presence of remodeling or loss of bone volume), and contributes 1 point to the S-score. For any patient, the maximal S-score will therefore be 36 (i.e., all areas affected) and the minimal score 0 (i.e., no erosions in hands and/or feet).

To confirm the usefulness of the simplified score to capture joint damage, we first used a sample of RA patients to compare its correlation with a reference score. A total of 139 RA patients with >2 years of follow-up since the diagnosis of the disease were collected, and the degree of joint damage was measured using the S-score and the Sharp–van der Heijde Score (SHS) as the reference score [9]. Although it requires expert training and a substantial amount of time, the SHS is clearly one of the most used scores to quantify joint damage in RA. A highly significant positive correlation (α <0.001) between both scores was considered a validation of the usefulness of the S-score to quantify the level of joint destruction in RA patients. In this group of patients we found a highly significant correlation between the S-score and the SHS reference score (*P* = 5.42 × 10^−20^, *r*^*2*^ (95 % confidence interval (CI)) = 0.67 (0.57–0.75)), and therefore it was considered an informative measure of joint damage.

#### IL6R tagging SNP selection

**Fig 1:**
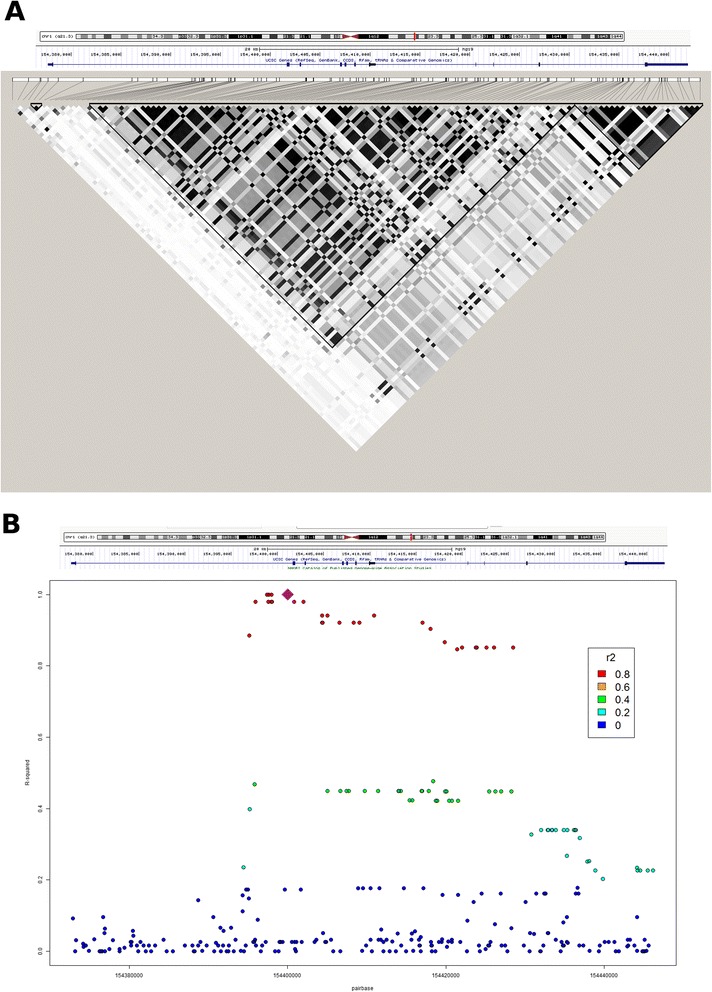
LD pattern at the IL6R locus. **a** Identification of the main haplotype blocks. Using Hapmap on 1000 Genomes Project data, we identified two main haplotype blocks covering most of the *IL6R* sequence. The 5′ block covers an ~38 kb region (154,432,622–154,394,417 bp), and the 3′ block covers an ~6 kb IL6R sequence (154,432,877–154,439,865 bp). Using Haploview software (v 4.2; Broad Institute of MIT/Harvard University, Cambridge, Massachussetts, USA), we searched for tagging SNPs within each haplotype block. **b** LD pattern associated with SNP rs4845618. LD (*r* ^2^, *y* axis) between SNPs in the IL6R locus and rs4845618 (purple diamond), the SNP most significantly associated with joint damage in RA. LD estimation was obtained from Caucasian European data from Hapmap samples generated by the 1000 Genomes Project [10]. SNPs are colored according to the LD level with rs4845618 (see legend). Markers with high LD (i.e. *r* ^2^ >0.8) are mapped within the transcribed region of IL6R

TagSNP identification for *IL6R* was performed using the Tagger method implemented in Haploview software (v 4.2; Broad Institute of MIT/Harvard University, Cambridge, Massachussetts, USA). For this objective, the genotype data from *IL6R* transcribed region (154,377,669–154,441,926 base pairs (bp) in chromosome 1) were analyzed, including 5000 bp flanking regions at the 5′ and 3′ ends of the gene. To identify the best tagging SNPs, high-density genotyping data from the Hapmap Caucasian European population samples generated 1000 Genomes Project (1KG) were used [10]. Within the *IL6R* coding region, a total of four splice donor/acceptor variants, three stop gaining variants, three frameshift variants, and 87 missense variants have been identified in the *IL6R* sequence. Most of these variants, however, are rare (<1 % frequency) or have only been identified at the single individual level (94.3 % variants). Haplotype block analysis identified three main haplotype blocks (Fig. 1a), with a relatively high linkage disequilibrium (LD) between them (*r*^*2*^ >0.7). Pairwise tagging SNPs were selected (minor allele frequency >0.05, pairwise *r*^*2*^ >0.8; Additional file 1). TagSNPs tagging <2 SNPs were not selected. A total of five tagSNPs were finally selected that tagged 101 SNPs identified in the *IL6R* locus.

#### SNP genotyping

Genomic DNA was isolated from venous blood samples obtained from RA patients using the Chemagic Magnetic Separation Module I (PerkinElmer, Waltham, Massachusetts, USA). SNP genotyping was performed using the TaqMan® genotyping platform (Life Technologies, Carlsbad, California, USA). The TaqMan® assays used to genotype IL-6R tagSNPs were: C_____30047_10 (rs4845618), C__26292294_10 (rs4453032), C__45267034_10 (rs4845374), C__11258914_10 (rs6698040), and C__27968911_10 (rs4379670). Thermal cycle conditions were as follows: 50 °C for 2 minutes and 95 °C for 10 minutes, followed by 40 cycles of 92 °C for 15 seconds and 60 °C for 1 minute. All PCR and endpoint fluorescent readings were performed using an ABI PRISM 7900 HT sequence detection system (Life Technologies). The genotyping error was estimated by genotyping 20 % of the samples in duplicate (error <1 %).

#### Statistical analysis

The association between *IL6R* tagging SNPs and joint damage in RA was tested using linear regression with an additive model. Using the linear modeling framework allowed us to control for relevant covariates such as age, years of disease duration, gender, anti-citrullinated protein antibodies (ACPA) and rheumatoid factor (RF) status. All statistical tests were performed using the R statistical software version 3.0.1 [11]. Multiple test correction was performed using the false discovery rate (FDR) procedure.

The meta-analysis of the association results from the discovery and replication cohorts was performed using METAL software [12]. This method uses an inverse variance strategy to weight the association statistics according to their effect size estimates (i.e., beta coefficients from linear regression) and their standard errors from the discovery and validation cohorts of patients.

#### Haplotype analysis

*IL6R* haplotype blocks were defined using the model described by Gabriel et al. [13]. Haplotypes were assigned to each individual using PLINK software (version 1.07; Center for Human Genetic Research (CHGR), Massachusetts General Hospital (MGH), and the Broad Institute of Harvard and MIT, Boston, Massachusetts). Analyses of the haplotypes were performed using the same linear regression analysis approach as for the single-marker association analysis.

## Results

### Discovery phase

A total of 527 RA patients were analyzed for association between *IL6R* tag SNPs and joint destruction. We found a highly significant association between SNP rs4845618 and the level of joint damage in the discovery cohort of patients (*P* = 0.0052, β (95 % CI) = −1.09 (−0.33 to −1.85); Table 1). This association was still significant after multiple testing correction (*P*_FDR_ = 0.026). We also found a significant association between SNP rs4845374 (*P* = 0.02, β (95 % CI) = 1.14 (0.18–2.10); Table 1). After multiple test correction this SNP was almost significant (*P*_*FDR*_ = 0.050). Both associated *IL6R* SNPs were in low LD between them (*r*^*2*^ = 0.14).

**Table 1:** Association of *IL6R* locus SNPs with the severity of joint damage in RA

				Discovery phase		Replication phase		
SNP	Base pair	Minor allele	MAF	β^a^ (95 % CI)	*P* value	β^a^ (95 % CI)	*P* value	Meta-analysis *P* value^b^
rs4845618	154,400,015	*G*	0.44	−1.09 (−1.85 to −0.33)	0.0052	−1.01 (−1.74 to −0.28)	0.007	0.00011
rs4453032	154,414,086	*G*	0.40	0.25 (−0.51 to 1.01)	0.51	–	–	–
rs4845374	154,426,947	*A*	0.17	1.14 (0.18–2.10)	0.02	0.95 (0.04–1.85)	0.04	0.0021
rs6698040	154,432,948	*T*	0.21	−0.79 (−1.75 to 0.17)	0.11	–	–	–
rs4379670	154,439,865	*T*	0.16	−0.64 (−1.72 to 0.44)	0.25	–	–	–

*CI* confidence interval, *IL6R* interleukin-6 receptor gene, *MAF* minor allele frequency, *RA* rheumatoid arthritis, *SNP* single nucleotide polymorphism

^a^Correlation coefficient estimate, indicating the fold difference in joint damage in the presence of the minor allele (i.e., negative coefficients indicate a decrease in joint damage, positive coefficients indicate an increase of joint damage)

^b^ *P* value estimated from the meta-analysis of the significance and effect sizes of discovery and replication cohorts

An exploratory analysis using different genetic models (i.e., genotypic, dominant, and recessive) did not find strong evidence for an alternative genotype to phenotype association for *IL6R* SNPs (Table S1 in Additional file 1). Only under the dominant model was there a modest improvement over the original statistical significance for the two SNPs rs4845618 and rs4845374. We also explored the influence of autoantibody status on the association of the five *IL6R* tagSNPs with joint damage (Table S2 in Additional file 1). In this case, stratifying according to ACPA or RF status did not increase the statistical significance of IL6R SNPs with this disease severity trait. Haplotype analysis using the two associated SNPs identified a significant association between haplotypes *GT* and *TA* with the S-score (β = −1.12, *P* = 0.0037 and β = −1.28, *P* = 0.0099, respectively; Table 2). Both haplotypes were significantly associated with joint damage after multiple test correction (*P*_*FDR*_ = 0.011 and *P*_*FDR*_ = 0.015 for *GT* and *TA* haplotypes, respectively). Using all five tagSNPs to test for haplotype association, we found less significant association. From the five estimated haplotypes, only one showed a nominally significant association (*P* = 0.019; Table 2). According to these results only, the significant SNPs rs4845618 and rs4845374 were tested in the independent patient dataset.

**Table 2:** Haplotype association results between *IL6R* locus SNPs with the severity of joint damage in RA

		*P* value^c^	
Haplotype^a^	SNPs^b^	Discovery phase	Replication phase
*TA*	rs4845618|rs4845374	0.0099	0.052
*GT*	rs4845618|rs4845374	0.0037	0.0058
*TT*	rs4845618|rs4845374	0.38	0.023
*GATTT*	rs4845618|rs4453032|rs4845374|rs6698040|rs4379670	0.31	–
*GATTA*	rs4845618|rs4453032|rs4845374|rs6698040|rs4379670	0.14	–
*TAACA*	rs4845618|rs4453032|rs4845374|rs6698040|rs4379670	0.02	–
*TGTCA*	rs4845618|rs4453032|rs4845374|rs6698040|rs4379670	0.27	–
*GATCA*	rs4845618|rs4453032|rs4845374|rs6698040|rs4379670	0.12	–

*IL6R* interleukin-6 receptor gene, *RA* rheumatoid arthritis, *SNP* single nucleotide polymorphism

^a^Imputed haplotype according to the E–M algorithm implemented in PLINK (version 1.07; Center for Human Genetic Research (CHGR), Massachusetts General Hospital (MGH), and the Broad Institute of Harvard and MIT, Boston, Massachusetts)

^b^List of IL6R tagSNPs used to estimate the haplotypes

^c^Statistical significance after testing the association of the estimated haplotype with the level of joint damage. Only haplotypes with a frequency >1 % were tested for association

### Replication phase

In the replication phase a total of 705 RA patients were analyzed for validation of the association between *IL6R* SNPs and the S-score. We significantly validated the association of both SNPs rs4845618 and rs4845374 (*P* = 0.007, β (95 % CI) = −1.01 (−1.74 to −0.28), and *P* = 0.04, β (95 % CI) = 0.95 (0.04–1.85), respectively) (Table 1). In both SNPs, the direction of the effect (i.e., positive/negative β coefficient) was the same as in the discovery cohort. Both polymorphisms were significantly associated with joint damage after multiple test correction (*P*_FDR_ = 0.014 and *P*_FDR_ = 0.040 for rs4845618 and rs4845374, respectively).

Haplotype analysis with the two validated SNPs replicated the association between *GT* and *TA* haplotypes with the S-score identified in the discovery phase (*P* = 0.0058 and *P* = 0.052, respectively; Table 2). In this case, only *GT* haplotype association withstood multiple test correction (*P*_*FDR*_ = 0.017 and *P*_*FDR*_ = 0.078 for *GT* and *TA* haplotypes, respectively).

After performing the meta-analysis of the two SNPs tested in both the discovery and replication datasets, the statistical significance of the association of both *IL6R* markers with joint damage was found to increase (*P* = 0.00011 and *P* = 0.0021 for rs4845618 and rs4845374, respectively) (Table 1). The association was strongest when considering the two SNPs as a haplotype (*GT* haplotype, *P* = 0.0058, meta-analysis *P* = 6.65 × 10^−8^).

## Discussion

The key measure of severity in RA is the presence of joint erosion as determined by radiological examination. The IL-6 signaling pathway is essential in the proinflammatory network that contributes to tissue destruction in RA. Blocking a key member of this network, the IL-6 receptor has proven highly efficacious in the inhibition of joint damage [5]. We have performed a candidate-gene association study to test the association of genetic variation at *IL6R* with the level of erosions in RA.

To date, three main mRNA isoforms of *IL6RA* have been described, with 10, 9 and 7 exons (mRNA isoforms 1, 2 and 3, respectively). We have found a strong association between a SNP in the first intron common to all isoforms and joint damage (SNP rs4845618, *P* = 0.00011), and a moderate association of a SNP in the sixth intron of isoforms 1 and 2 (rs4845374, *P* = 0.0021). After combining both variants rs4845618 and rs4845374 as a haplotype, the association with the level of joint damage in RA was found to be stronger (*P* = 6.45 × 10^−5^). This result suggests that neither of the two associated variants are the causal polymorphism that influences joint destruction, and instead they are proxies for another polymorphism. The LD pattern associated with the most strongly associated SNP, rs4845618, suggests that the causal polymorphism lies within the transcribed sequence of *IL6R* (Fig. 1b). In this region lies SNP rs2228145, a variant that was recently found to have profound functional implications in *IL6R* expression [14]. Importantly, this variant has also been associated with RA risk [14, 15]. However, the LD between rs4845374 and the SNP associated with joint damage rs4845618 is moderate in the Caucasian European population, like our Spanish RA patient cohorts (*r*^*2*^ = 0.44; 1KG data). Furthermore, the marker tagging rs2228145, SNP rs4453032 (*r*^*2*^ = 0.91), showed no evidence of association with the level of joint erosions in RA (*P* = 0.51; Table 1). These results therefore suggest that the genetic variant associated with joint erosions in RA is not the same variant associated with disease risk. Future studies using next-generation sequencing analysis will be valuable to identify the precise variation at the IL6R locus that increases the risk of developing joint erosions at the *IL6R* locus.

In most human complex traits, the vast majority of genetic associations are being identified outside coding regions [16], and therefore the functional link is generally difficult to identify. In the present study, the SNP showing the strongest association to joint damage, rs4845618, is an intronic SNP of *IL6R*. Evaluating publicly available results on tissue expression quantitative loci (eQTL) analysis [17], there is evidence supporting rs4845618 being a highly significant cis-eQTL for *IL6R* expression in whole blood (*P* = 1.1 × 10^−16^; data not shown). Also, analyzing the most recent regulatory and epigenetic evidence generated from large international consortia such as the Roadmap Epigenomics project [18], there is strong regulatory evidence associated with this SNP (Table S3 in Additional file 1). In particular, the rs4845618 SNP region is associated with regulatory activity in more than 50 different human cell types, including CD4^+^ T cells. Together these results support the present genetic association with joint damage being likely to influence *IL6R* regulatory activity.

Tocilizumab is a monoclonal antibody against IL6R, prevents downstream IL-6 signaling and has been shown to significantly reduce the signs and symptoms of RA. In particular, there is increasing evidence that IL6R blockade prevents structural joint damage [19]. IL-6 is a pleiotropic inflammatory cytokine and therefore many biological mechanisms can explain the efficacy of this treatment [3]. From these mechanisms, the inhibition of inflammatory osteoclastogenesis after IL6R blocking [20] is clearly the most direct biological process influencing the level of joint destruction in RA. There is recent suggestive evidence that variants at *IL6R* are associated with the clinical response to tocilizumab [21]. While these results are still preliminary, they provide additional support for the importance of variation at this gene with disease proinflammatory activity. Future studies analyzing the association between SNPs in the present study with the response to tocilizumab are possible.

In the present study we prioritized the selection of the best tagging SNPs of *IL6R* variation over previously published variants, in order to capture most of the common variation in this gene. For example, *IL6R* SNP rs8192284 (now merged into SNP rs2228145) – which had been previously associated with disease activity in RA [22] – was not selected for genotyping. Importantly, however, rs8192284 is in very high LD with the genotyped SNP rs4453032 (*r*^*2*^ = 0.91, 1000 Genomes Project data on a Caucasian European population). As shown in the discovery stage, there is no evidence for association between SNP rs4453032 and joint damage in RA (*P* = 0.51; Table 1). The high LD between rs8192284 and rs4453032 therefore supports the lack of association of this variant with joint damage in RA. This result confirms that the objective selection of the most informative markers is a powerful approach to identify the relevant genetic variation associated with disease severity in RA.

One potential limitation of this study is that using a simplified score might have reduced the statistical power of the association. It is possible that using a more standard score such as the SHS we would have better assessed joint damage in the RA cohorts, and therefore found a more significant association with the genetic variation at the *IL6R* locus. Nonetheless, the significant association of *IL6R* found both in the discovery cohort and in the validation cohort strongly supports the usefulness of the S-score to capture the essential features of this trait in RA. While the loss of information from not using a reference score like SHS would be unacceptable in clinical studies such as treatment efficacy analysis, this simplified erosion score could be a practical tool in those instances like genetic studies in which a large number of patient data must be collected from multiple centers and there is no possibility to perform more complex scores of joint damage.

## Conclusions

In this study we have identified and replicated variation at *IL6R* associated with the level of joint damage in RA. Further functional studies at the transcriptional and protein level are needed to characterize the precise molecular mechanism influenced by this genetic variation.

## Additional file

Additional file 1: Table S1. Presenting the association of *IL6R* locus SNPs with joint damage in the discovery stage under alternative genetic models, **Table S2**. Presenting the association of *IL6R* locus SNPs with joint damage in the discovery stage according to ACPA or RF status, and **Table S3**. Presenting the enhancer histone marks associated with the *IL6R* rs4845618 SNP identified in 111 reference epigenomes from the Epigenome Roadmap Project.

## Abbreviations

ACPA: Anti-citrullinated protein antibodies
bp: Base pairs
CI: Confidence interval
eQTL: Expression quantitative loci
FDR: False discovery rate
IL: Interleukin
*IL6R*: Interleukin-6 receptor gene
IL6R: Interleukin-6 receptor protein
LD: Linkage disequilibrium
*r*^2^: Linkage disequilibrium statistic
RA: Rheumatoid arthritis
RF: Rheumatoid factor
SNP: Single nucleotide polymorphism
SHS: Sharp–van der Heijde Score.
