# Supplementary material for: Variation at interleukin-6 receptor gene is associated to joint damage in rheumatoid arthritis
Source: Arthritis Res Ther. 2015 Sep 4;17(1):242. doi: 10.1186/s13075-015-0737-8 (PMC4560075; doi:10.1186/s13075-015-0737-8)
Supplement: Additional file 1: Table S1. — Presenting the association of IL6R locus SNPs with joint damage in the discovery stage under alternative genetic models, Table S2. Presenting the association of IL6R locus SNPs with joint damage in the discovery stage according to ACPA or RF status, and Table S3. Presenting the enhancer histone marks associated with the IL6R rs4845618 SNP identified in 111 reference epigenomes from the Epigenome Roadmap Project. [file 13075_2015_737_MOESM1_ESM.pdf]

SUPPLEMENTARY MATERIAL

Supplementary Table 1. Association of *IL6R* locus SNPs with joint damage in the discovery stage under alternative genetic models.

SNP	Basepair	Genetic Model	*P*-value
rs4845618	154400015	Genotypic	0.012
		Dominant	0.0025*
		Recessive	0.16
rs4453032	154414086	Genotypic	0.37
		Dominant	0.38
		Recessive	0.80
rs4845374	154426947	Genotypic	0.0020*
		Dominant	0.0027*
		Recessive	0.59
rs6698040	154432948	Genotypic	0.21
		Dominant	0.74
		Recessive	0.08
rs4379670	154439865	Genotypic	0.60
		Dominant	0.99
		Recessive	0.21

*****Significance *P-values* showing modest improvement over original genetic association under the additive model.

Supplementary Table 2. Association of *IL6R* locus SNPs with joint damage in the discovery stage according to ACPA or RF status.

SNP	Basepair	Autoantibody Group	*P*-value
rs4845618	154400015	ACPA+	0.0096
		ACPA-	0.26
		RF+	0.024
		RF-	0.08
rs4453032	154414086	ACPA+	0.31
		ACPA-	0.62
		RF+	0.42
		RF-	0.97
rs4845374	154426947	ACPA+	0.094
		ACPA-	0.055
		RF+	0.16
		RF-	0.026
rs6698040	154432948	ACPA+	0.16
		ACPA-	0.47
		RF+	0.16
		RF-	0.44
rs4379670	154439865	ACPA+	0.18
		ACPA-	0.98
		RF+	0.21
		RF-	0.84

None of the 5 IL6R tagSNPs showed an improvement of the association with joint damage after stratifying for ACPA or RF status.

Supplementary Table 3. Enhancer histone marks associated with SNP *IL6R* rs4845618 SNP identified in 111 reference epigenomes from the Epigenome Roadmap Project.

Cell ID	Cell description	State (25-state HMM)
**COL.SMUS**	Colon Smooth Muscle	2_TssF
**R.MUC29**	Rectal Mucosa.Donor 29	6_TssD2
**PFM.2**	Penis Foreskin Melanocyte Primary Cells.Donor skin02	2_TssF
**CD4.NP**	CD4 Naive Primary Cells	11_EnhWk1
**CD4.MP**	CD4 Memory Primary Cells	11_EnhWk1
**CCIP.LSTP**	CD4+ CD25- IL17+ PMA-Ionomcyin stimulated Th17 Primary Cells	11_EnhWk1
**SK.MUS**	Skeletal Muscle	13_EnhA
**ST.MUC**	Stomach Mucosa	13_EnhA
**LIV.A**	Adult Liver	13_EnhA
**PFK.3**	Penis Foreskin Keratinocyte Primary Cells.Donor skin03	11_EnhWk1
**BR.H35**	Breast vHMEC.Donor RM035	11_EnhWk1
**PFM.3**	Penis Foreskin Melanocyte Primary Cells.Donor skin03	11_EnhWk1
**DUO.SMUS**	Duodenum Smooth Muscle	13_EnhA
**ESO**	Esophagus	13_EnhA
**GAS**	Gastric	13_EnhA
**LV**	Left Ventricle	13_EnhA
**PFK.2**	Penis Foreskin Keratinocyte Primary Cells.Donor skin02	11_EnhWk1
**SK.MUS63**	Skeletal Muscle.Donor 63	13_EnhA
**ADI.NUC**	Adipose Nuclei	13_EnhA
**COL.MUC32**	Colonic Mucosa.Donor 32	11_EnhWk1
**R.MUC31**	Rectal Mucosa.Donor 31	13_EnhA
**SK.MUS62**	Skeletal Muscle.Donor 62	13_EnhA
**ADI.MSC**	Adipose Derived Mesenchymal Stem Cell Cultured Cells	11_EnhWk1
**BR.MYO**	Breast Myoepithelial Cells	11_EnhWk1
**BN.GM2**	Brain Germinal Matrix.Donor HuFGM02	11_EnhWk1
**HRT.FE**	Fetal Heart	11_EnhWk1
**DUO.MUC61**	Duodenum Mucosa.Donor 61	13_EnhA
**PFM.1**	Penis Foreskin Melanocyte Primary Cells.Donor skin01	13_EnhA
**BN.ITL**	Brain Inferior Temporal Lobe	11_EnhWk1
**CCCRA.NP**	CD4+ CD25- CD45RA+ Naive Primary Cells	13_EnhA
**PANC**	Pancreas	13_EnhA
**H1.DMSC**	H1 Derived Mesenchymal Stem Cells	13_EnhA
**IMR90**	IMR90 Cell Line	9_TxEnhG1
**CD34.MBP1562**	Mobilized CD34 Primary Cells.Donor RO 01562	11_EnhWk1
**BN.MFL**	Brain Mid Frontal Lobe	13_EnhA
**PFF.1**	Penis Foreskin Fibroblast Primary Cells.Donor skin01	11_EnhWk1

**CD15.P**	CD15 Primary Cells	9_TxEnhG1
**SPL**	Spleen	11_EnhWk1
**BN.HM150**	Brain Hippocampus Middle.Donor 150	11_EnhWk1
**LNG.FE**	Fetal Lung	11_EnhWk1
**BN.CC**	Brain Cingulate Gyrus	11_EnhWk1
**BN.SN**	Brain Substantia Nigra	13_EnhA
**PANC.I**	Pancreatic Islets	14_Enh
**BN.AC**	Brain Anterior Caudate	13_EnhA
**CCIP.LSMPTP**	CD4+ CD25- IL17- PMA-Ionomycin stimulated MACS purified Th Primary Cells	9_TxEnhG1
**CCC.TREGP**	CD4+ CD25+ CD127- Treg Primary Cells	9_TxEnhG1
**ST.SMUS28**	Stomach Smooth Muscle.Donor 28	13_EnhA
**R.SMUS**	Rectal Smooth Muscle	13_EnhA
**BN.AG**	Brain Angular Gyrus	13_EnhA
**CD34.C**	CD34 Cultured Cells	11_EnhWk1
**CHON.BMMSC**	Chondrocytes from Bone Marrow Derived Mesenchymal Stem Cell Cultured Cells	11_EnhWk1
**CC.TPC**	CD4+ CD25- Th Primary Cells	13_EnhA
**MUS.SC**	Muscle Satellite Cultured Cells	11_EnhWk1
**MSC.ADIPC**	Mesenchymal Stem Cell Derived Adipocyte Cultured Cells	11_EnhWk1
**PFF.2**	Penis Foreskin Fibroblast Primary Cells.Donor skin02	14_Enh

In 55 out of 111 epigenomes from different human cell subtypes there is evidence of regulatory activity for the genomic region harboring SNP rs4845618. The data retrieval was performed using HaploReg v3 (http://www.broadinstitute.org/mammals/haploreg/haploreg_v3.php) a software tool developed to explore genomic annotations on the noncoding genome and therefore identify new mechanistic hypotheses of non-coding variants on clinical phenotypes. TssP: TSS_poised; TssF: TSS_flanking_more_upstream; TssA: TSS_active; TssWk:TSS_weak; TssD1: TSS_flanking_downstream; TssD2: TSS_flanking_more_downstream; Tx: Transcription; TxWk: Transcription_weak; TxEnhG1: Transcription Enhancer-like; TxEnhG2: Transcription Enhancer-like; EnhWk1: Enhancer_weak; EnhWk2: Enhancer_weak; EnhA: Enhancer_active; Enh: Enhancer_active_with_weakK4me1_strong_K27ac; EnhP: Enhancer_poised; ReprPCWk: Repressed_polycomb_weak; ReprPC: Repressed_polycomb; K9K27me3: H3K9me3_K27me3; ZNF: Zinc_finger_genes_H3K36me3_K9me3; HetRpts: Heterochromatin_at_repeats; Het: Heterochromatin.
